# Supplementary material for: Comparative Evaluation of Bleomycin- and Collagen-V-Induced Models of Systemic Sclerosis: Insights into Fibrosis and Autoimmunity for Translational Research
Source: Int J Mol Sci. 2025 Mar 14;26(6):2618. doi: 10.3390/ijms26062618 (PMC11942454; doi:10.3390/ijms26062618)
Supplement: Supplementary file 1 [file ijms-26-02618-s001.zip › ijms-3508614-supplementary.pdf]

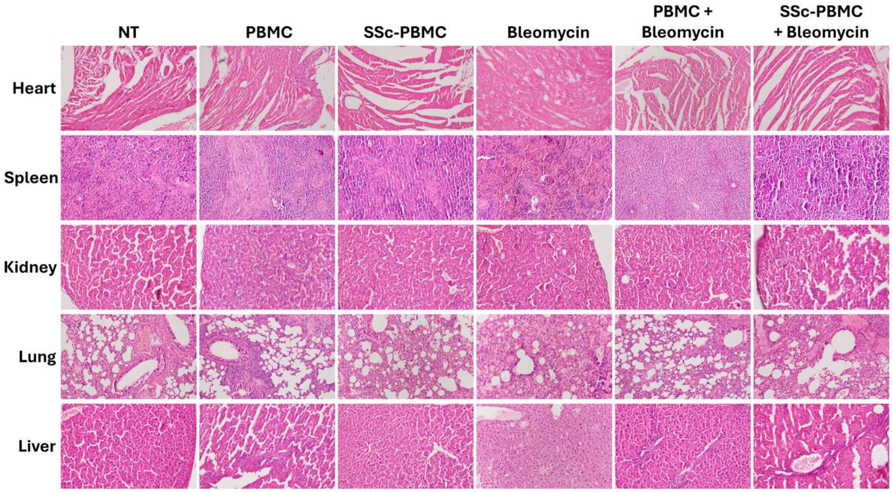

Supplementary Figure S1. Overview of hematoxylin-eosin staining in the bleomycin-based model experiment. Maginfication was 20×.

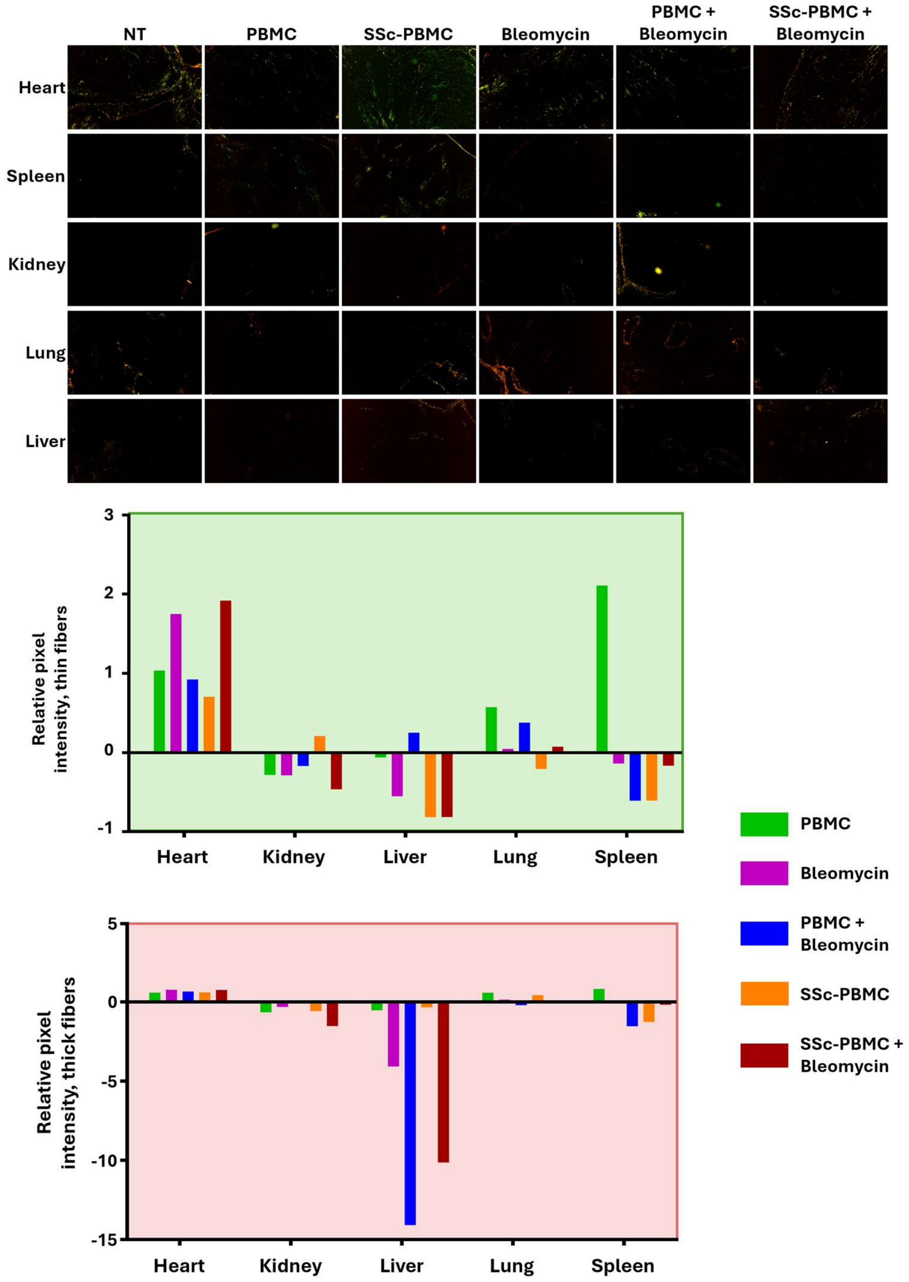

Supplementary Figure S2. Picrosirius Red staining – Bleomycin experiment. A) Overview of representative micrographs from each treatment groups. Images shown here are identicaly enhanced for better visibility. B-C) Meassured green (B) and red (C) pixel intensities, corresponding to thin (B) and thick (C) collagen fibers, shown as fold-wise comparison to the non-treated ("NT") group's values in the respective organ. Maginfication was 20×.

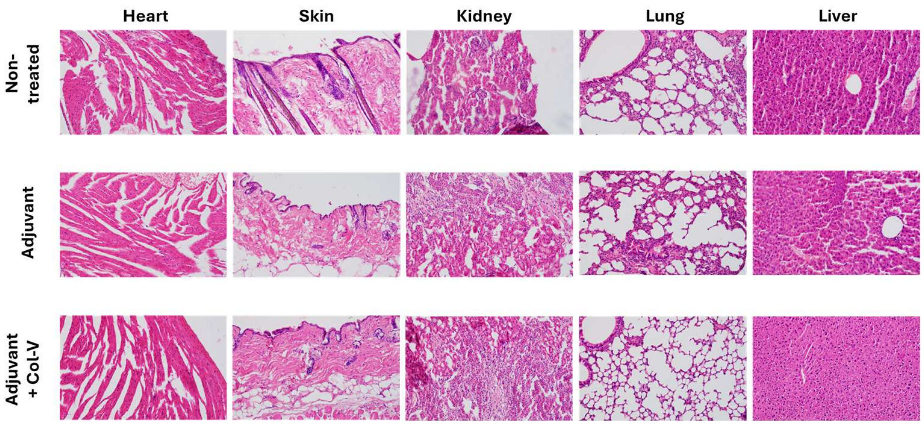

Supplementary Figure S3. Hematoxylin-eosin staining – Collagen-V experiment. Overview of representative micrographs from each treatment group. Maginfication was 20×.

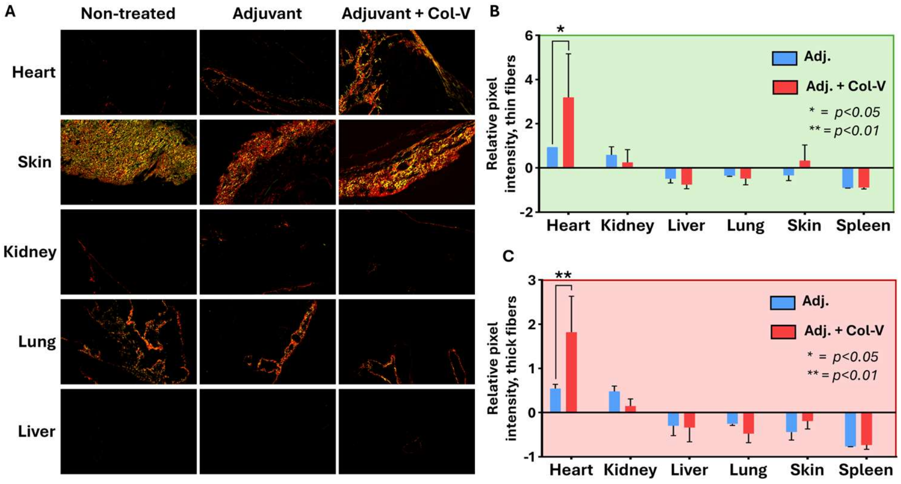

Supplementary Figure S4. Picrosirius Red staining – Collagen-V experiment. A) Overview of representative micrographs from each treatment groups. Images shown here are identicaly enhanced for better visibility. B-C) Meassured green (B) and red (C) pixel intensities, corresponding to thin (B) and thick (C) collagen fibers, shown as fold-wise comparison to the non-treated ("NT") group's values in the respective organ. Treatment group means compared with two-tailed t-test, error bars representing SEM. Maginfication was 20×.

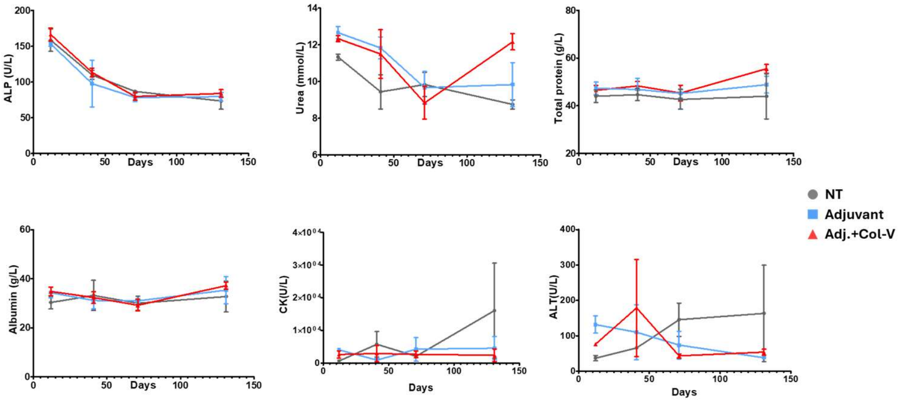

Supplementary Figure S5. Routine clinical chemistry serum test results – Collagen-V experiment. Diagnostic values determined for each mice at each timepoint, n=3 per group. Error bars representing SD.
